# Study of the Association of Insecure Attachment With the Dehumanization and Self-Dehumanization of Patients Hospitalized With Psychotic Disorder and Organic Disease

**DOI:** 10.7759/cureus.21445

**Published:** 2022-01-20

**Authors:** Dimitra Lekka, Clive Richardson, Anna Madoglou, Konstantina Orlandou, Vasileia Arachoviti, Vassia I Karamanoli, Aikaterini Roubi, Constantinos Togas, Athanasios Tsaraklis, Anastasios Stalikas

**Affiliations:** 1 Department of Psychiatry, Thoracic Diseases General Hospital Sotiria, Athens, GRC; 2 Department of Economic and Regional Development, Panteion University of Social and Political Sciences, Athens, GRC; 3 Department of Psychology, Panteion University of Social and Political Sciences, Athens, GRC; 4 Psychology, Hellenic Military Academy, Athens, GRC; 5 Psychology, Independent Researcher, Megalopolis, GRC; 6 Department of Dermatology, Thoracic Diseases General Hospital Sotiria, Athens, GRC

**Keywords:** insecure attachment, organic disease, psychotic disorder, self-dehumanization, dehumanization

## Abstract

Introduction

Dehumanization is the phenomenon that concerns the non-attribution of humanness to other human beings and has two dimensions, animalistic and mechanistic. The aim of the present study is to study dehumanization and self-dehumanization in patients with psychosis and organic disease.

Methods

The sample consisted of 200 people who were hospitalized in Athens, Greece, in 2017. Participants were asked to answer the dehumanization questionnaire, the mechanistic self-dehumanization scale, the human nature and human uniqueness characteristics questionnaire, and the adult attachment questionnaire.

Results

It was found that patients with organic disease and patients with psychosis do not perform mechanistic and animalistic dehumanization of themselves. Still, it seems that insecure attachment (anxiety and obsession) contributes positively to their mechanistic dehumanization and negatively to their mechanistic self-dehumanization. From the insecure attachment, only the dimensions of stress and obsession affect the mechanistic dehumanization.

Conclusions

It is important to take measures to train specialists in the hospital so that they can establish a safe therapeutic relationship with the patient so that patients will not resort to dehumanization and self-dehumanization as a defense against the stress of hospitalization.

## Introduction

According to Haslam's model [1], dehumanization is the phenomenon of the non-attribution of human qualities to other human beings. Humanness has two different dimensions: one represents qualities that are unique to the human species (human uniqueness), and the other represents those qualities that are essential or fundamental to humans (human nature). In a complete model of dehumanization, these separate forms of dehumanization correspond to the denial of the two dimensions of people with specific types of non-human entities e.g. animals (animalistic dehumanization) and machines (mechanistic dehumanization) [1]. Dehumanization, according to Gray et al. [2] is the denial of the mind capable of agency (e.g., intention and free will) and experience (e.g., emotion and movement).

Dehumanization is not just about perceptions of others but also about perceptions of oneself. This may be the result of harmful treatment by others, or it may be caused by one's own harmful behavior [3]. Self-dehumanization also has consequences for emotions and behavior. It is associated with dissuasive self-awareness, cognitive degenerative states, and feelings of shame, guilt, sadness, and anger. Self-dehumanization can also motivate behavior aimed at recovery, perhaps in an attempt to regain the lost humanness [4].

Dehumanization and self-dehumanization are phenomena that have serious consequences. These possible consequences can be divided into four broad groups. Dehumanized perceptions of individuals or groups can reduce emotional behavior toward them [5]. On the other hand, people in a secure attachment type use emotion management strategies that minimize stress and anxiety while emphasizing positive emotions. Trust is an important component in healthy relationships. The ability of the individual to detect the emotions and tensions of emotion in the other person's face could increase this confidence [6]. People in an insecure type of relationship follow emotion regulation strategies that focus on negative emotions and experience interpersonal or other situations as more stressful - emotion type or tend to suppress their emotions and not to remember emotional information - type of avoidance [7,8]. In this regard, relationships with close others should promote the appreciation of all people as fully human, rather than increase the distance between oneself and others and cause humanity to deny them [9]. Research findings show that people who felt interpersonally safe were less prone to dehumanizing other people and, therefore, less likely to treat them harshly. Interpersonal security promoted by safe and warm social interactions leads to a greater rendering of humanness to others. Feelings of interpersonal security may be inherently intertwined in approaching others who provide love and support [9].

The purpose of this study is to investigate the dehumanization and self-dehumanization aspects in hospitalized patients, with a special focus on patients with psychotic disorders and organic diseases. Thus, the following hypotheses were formulated:

H1: Psychotic patients dehumanize less than patients with organic disease.

H2: These psychotic patients are mechanically dehumanized more than patients with organic disease.

H3: Patients with insecure attachment are less self-dehumanized.

## Materials and methods

Sampling

Convenience sampling was used to select the patients who participated in the study. Sampling took place in Thoracic Diseases General Hospital Sotiria, based in Athens, Greece, in which the first author works. Patients with mental illness were drawn from the hospital's psychiatric clinic and patients with organic disease from the nursing wards of the same hospital. Questionnaires were administered to patients through a personal approach by the researcher to the nursing wards where they were treated. The total sample was 200 patients. All questionnaires were valid.

Measures

The questionnaires were completed by the patients, in 2017, at Thoracic Diseases General Hospital Sotiria. Before completing the questionnaires, the interviewed subjects were asked to provide basic demographic data (gender, age, educational level).

Dehumanization questionnaire

The dehumanization questionnaire is based on the model of Haslam [1], consisting of eight pairs of characteristics (e.g., distant/cold vs. warmth), which must be attributed to the patient. It has two dimensions: mechanistic dehumanization and animalistic dehumanization. Statements 1-4 (e.g., distant/cold, vs. warmth) constitute a measure of mechanistic dehumanization, and statements 5-8 (e.g., instinctive vs. rational) measure animalistic dehumanization. The respondent rates each statement on a nine-point Likert scale where "1" corresponds to the attribute on the left side of the scale (e.g., without autonomy and will) and "9" to the attribute on the right side of the scale (e.g., autonomous and voluntary). The questionnaire has been translated and adapted into Greek by Sakalaki [10,11]. In the present study, Cronbach’s α internal consistency index was α=0.76.

Mechanistic self-dehumanization scale

Inspired by the models of Haslam [1] and Gray et al. [2], the mechanistic dehumanization scale [10] is a questionnaire consisting of 14 statements that measure the rating of human nature traits in the self (e.g., "I don't usually operate in an emotional way"). The statements are rated on a nine-point Likert scale (from 1 - strongly disagree to 9 - strongly agree), stating the degree of agreement with each statement. The higher rating refers to the greater self-dehumanization. The questionnaire was negatively correlated with the questionnaire of human nature and uniquely human characteristics, which were inspired by the model of Haslam [1]. The maximum score that can be given is 126. The scale has been used in the literature to assess self-humanization, which makes it suitable for this study [10,11]. In the present study, Cronbach’s α internal consistency index was α=0.79.

Human nature (HN) and human uniqueness (HU) characteristics questionnaire

The questionnaire consists of eight statements regarding the attribution of HN, HU characteristics to the self. It comprises two subscales with four sentences, each assessing the dimensions of human nature and human uniqueness. Propositions 1-4 constitute the HN subscale, and propositions 5-8 constitute the HU subscale. Statements 3-4 from the HN subscale, and statements 7-8 from the HU subscale have reverse coding. The rating is done on a nine-point Likert scale (from 1 - not at all to 9 - too much), which indicates the degree of agreement with the situation described in each statement. The higher the value, the greater the rating of the HN and HU attributed to the self. The maximum score that can be given per factor is 36. The questionnaire has been translated into Greek by Sakalaki [10]. In the questionnaire of the characteristics of human nature, human uniqueness, in the present research, Cronbach’s internal consistency was α=0.76.

Adult attachment questionnaire

The questionnaire consists of 36 statements that relate to romantic relationships, including marriage. It includes two subscales with 18 statements in each, which assess the two dimensions (anxiety/obsession and avoidance) of adult attachment. Based on these two dimensions, the respondents can be classified into four types of attachment. The respondent states whether he/she agrees with each of the 36 statements on a seven-point Likert scale (from 1 - strongly disagree to 7 - strongly agree). Odd-numbered statements make up the subscale of avoidance. Of these, 3, 7, 15, 17, 19, 21, 23, 25, 29, 31, 33, 35, have reverse coding. Even-numbered sentences form the subscale of stress/obsession. Of these, 18 and 22 have reverse coding. The average in each sub-scale gives the score of the bond in the two dimensions, and low values ​​of avoidance refer to safety [7,12]. The questionnaire Cronbach's internal consistency index was α=0.90.

Statistical analysis

The research was performed with a sequence of analyzes, utilizing Statistical Package for Social Sciences (SPSS) Base and SPSS Advanced Models (Edition 25, 2018; IBM Corp., Armonk, NY, USA). The analyses performed were t-test, regression, and two-way ANOVA. Effect sizes were calculated.

## Results

The final sample consisted of 200 patients treated in the surgery, psychiatry, and pathology clinics of the hospital. Of these, 101 patients (50.5%) were hospitalized in the Psychiatric Clinic and 99 patients (49.5%) in the Pathological and Surgical Clinics. The sample consisted of 80 men (40%) and 120 women (60%). The organic disease was in 46.5% of patients, and 53.5% of patients had a psychotic disorder. Regarding the educational level, 22 patients (11%) were graduates of primary school, 23 (11.5%) of secondary school, 49 (24.5%) of high school, 86 (43%) of university, and 20 (10%) had done postgraduate studies. In terms of age, 34 patients were 18-25 years old (17%), 40 patients were 26-35 years old (20%), 36 were 36-45 years old (18%), 51 were 46-60 years old (25.5%) and 39 patients were over 60 years old (19.5%). Occupationally, 7.5% of patients were undergraduates, 18% were self-employed, 9% were functionaries, 13.5% were private servants, 21% were retired, 5.5% were occupied with housekeeping, and 25.5% were unemployed (Table 1).

**Table 1:** Demographic characteristics of the sample (n=200), patients with psychotic disorder and with organic disease

Variables	Ν	%
Gender		
Men	80	40
Women	120	60
Age		
18-25	34	17
26-45	40	20
46-60	51	25.5
over 60	39	19.5
Educational level		
Primary school	22	11
Secondary school	23	11.5
High school	49	24.5
Bachelor's degree	86	43
Postgraduate studies	20	10
Occupation		
Undergraduate	15	7.5
Self-employed	36	18
Functionary	18	9
Private servant	27	13.5
Retired	42	21
Housekeeping	11	5.5
Unemployed	51	25.5
Patient		
Patients with organic disease	107	53.5
Patients with psychotic disorder	93	46.5

There was no statistically significant difference between patients with the organic disease and patients with the psychotic disorder in terms of mechanistic and animalistic dehumanization (p>0.05 in independent samples t-test).

Multiple regression analysis was used to examine whether the attachment dimensions (anxiety/obsession, avoidance) of hospitalized patients with psychotic disorder and patients with the organic disease were associated with their mechanistic dehumanization. From the review of the regression coefficients of the attachment dimensions, both avoidance (β=0.45, t=6.11, p<0.001), and anxiety/obsession were significantly associated with the mechanistic dehumanization of patients with the organic disease and patients with psychotic disorder (β=0.27, t=4.77, p<0.001). Cohen’s index was f^2^=0.24, indicating the medium magnitude of the effect (Table 2).

**Table 2:** Association of attachment dimensions (anxiety/avoidance) with the mechanistic dehumanization of patients with psychotic disorder and patients with organic disease in multiple regression analysis

Variable	B	Std Error	Beta	t	f^2^	p
Anxiety/obsession	0.27	0.09	0.17	4.77	0.24	<0.001>
Avoidance	0.45	0.09	0.34	6.11		<0.001>

Multiple regression was also used to examine whether the attachment dimensions (anxiety/obsession, avoidance) of hospitalized patients with psychotic disorder and patients with the organic disease were associated with their animalistic dehumanization. From the review of the regression coefficients, it was found that only anxiety/obsession was associated significantly with the animalistic dehumanization of patients (β=0.39, t=4.53, p<0.001), but not avoidance (p>0.05). Cohen's index f^2^=0.18 indicated a small effect.

The independent samples t-test showed no statistically significant difference in human nature (HN) trait between patients with psychotic disorder and patients with organic disease. However, there was a statistically significant difference in the uniquely human characteristics between patients with the organic disease and patients with psychotic disorder, t(198)=2.6, p=0.011. Cohen's d=0.36, indicating a small effect size. Patients with organic disease (M=6.9, SD=1.3) attributed the uniquely human characteristics to themselves significantly more than patients with psychotic disorder (M=6.4, SD=1.5).

In multiple regression, it was found that both anxiety/obsession (β=0.31, t=3.35, p=0.001) and avoidance were associated significantly and positively with the ratings of the human nature characteristics (β=0.55, t=5.68, p<0.001). Cohen's index f^2^=0.37 indicated a large effect size.

In multiple regression examining whether the attachment dimensions affect patients' attribution of the uniquely human to themselves, it was found that only the anxiety/obsession contributed significantly and positively (β=0.42, t=5, p<0.001), but not avoidance (p>0.05). Cohen's index f^2^=0.20 indicated that the effect size was medium.

The two groups of patients did not differ significantly in terms of mechanistic self-dehumanization (independent samples t-test, p>0.05). In multiple regression (Table 3), it was found that the attachment dimensions of both avoidance (β=-0.53, t=-7.56, p<0.001) and anxiety/obsession were associated significantly and negatively (β=-0.19, t=-2.89, p=0.004) with the mechanistic self-dehumanization of patients. Cohen’s index f^2^=0.52 indicated that the effect size was large.

**Table 3:** Association of attachment dimensions (anxiety/obsession-avoidance) with the mechanistic self-dehumanization of patients with psychotic disorder and patients with organic disease in multiple regression analysis

Variable	B	Std Error	Beta	t	f^2^	p
Anxiety/obsession	-0.19	0.07	-0.18	-2.89	0.52	0.004
Avoidance	-0.53	0.07	-0.48	-7.56		0.000

Gender did not seem to affect either mechanistic or animalistic dehumanization, nor the attribution of the human nature characteristics to the self and the human uniqueness characteristics to the self. Also, gender did not have a statistically significant effect on mechanistic self-dehumanization. Furthermore, age was not found to have a statistically significant effect on any of the above variables.

Finally, it was found that the level of education affected animalistic and mechanistic dehumanization, the attribution of the human nature and human uniqueness characteristic to the self, and the mechanistic self dehumanization of the patients.

## Discussion

In terms of dehumanization, we detected no differences between patients with psychotic disorder and patients with organic disease. This contradicts previous findings that patients with psychotic disorders are more dehumanized than patients with neurotic disorders [11]. However, it seems that the insecure attachment (anxiety/obsession, avoidance) of patients with psychotic disorder and patients with the organic disease is associated significantly positively with both their mechanistic and animalistic dehumanization. Psychosis is a major mental health problem and is characterized by high levels of interpersonal difficulties [13]. High levels of stress or avoidance in attachment relationships can be implicated in maintaining psychotic symptoms. Research findings show that both stress and avoidance are related to the overall level of interpersonal problems [14]. The findings of the relationship between avoidance and the negative symptoms of psychotic disorders support the cognitive models of schizophrenia which regard social withdrawal and emotional alleviation as methods of dealing with social anxiety. In schizophrenia, relationship difficulties are often portrayed as negative symptoms, such as social withdrawal. There is also a link between stress and avoidance with interpersonal problems, with specific correlations between stress and demanding behavior and also avoidance with interpersonal hostility.

High levels of avoidance are associated with therapeutic relationship difficulties [14-16]. The stress/obsession dimension is associated with physical illness [17]. The person feels the presence of the medical staff suffocating but is worried when they leave. The internal work model provides an almost constant distress signal and is the best means of maintaining closeness with staff. This leads people to believe that what is required during the illness is a way to keep the professional constantly by their side. This pattern of seeking care is characteristic of the individual whose inner sense of helplessness leads them to depend on others but finds the help of others insufficient [18]. Research findings show that the type of attachment is significantly associated with self-management and treatment outcomes of disease [19]. It is clear from the above that the dimensions of the anxiety/obsession and avoidance attachment in cases of disease where autonomy decreases and dependence on others increases mediate mechanistic and animalistic dehumanization.

In the present study, there were no differences in the dehumanization of patients between patients with psychotic disorder and patients with organic disease. However, it seems that the dimensions of attachment (anxiety/obsession and avoidance) have a significant negative effect on the dehumanization of patients with psychotic disorder and patients with organic disease. It seems that stress/obsession and avoidance have a protective effect on the dehumanization of the self and contribute to the humanity of the self. There were also no differences in the rating of the characteristics of human nature in themselves by the examined groups. The results show that patients with psychotic disorder and patients with organic disease equally attribute to themselves the characteristics of human nature, which are essential to humans and some of which are common to animals [1] and play a key role in self-perception [3]. But there were differences regarding the characteristics of human uniqueness. It seems that patients with organic disease attribute more of the features of human uniqueness to themselves than do patients with a psychotic disorder. This contradicts research findings that argue that patients with psychotic disorder humanize themselves more than neurotic people and attribute more positive emotions to themselves compared to neurotic patients [11]. The rating of fewer human uniqueness characteristics in patients with the psychotic disorder than in patients with the organic disease is likely to be the result of stigmatization [20-22], self-stigmatization [23], or the labels of mental illness [24-26]. It could also be a consequence of a cognitive focus on painful experiences that are critical to the perception of human life [10,27-29].

In terms of demographic characteristics, the gender and age of patients with psychotic disorder and patients with the organic disease did not affect their dehumanization (mechanistic and animalistic) or their self-dehumanization and the attribution of human nature and human uniqueness. In contrast, the educational level of patients with psychotic disorder and patients with organic disease affects both dehumanization (mechanistic and animalistic) as well as their self-dehumanization and the attribution of characteristics of human nature and human uniqueness to oneself. Specifically, our findings show that patients with the organic disease who are graduates of universities are mechanistically dehumanized more than patients who have a master's/doctoral degree. Psychotic patients who are elementary school graduates are mechanistically dehumanized more than those who are high school graduates. These results show differences in mechanistic dehumanization that for patients with organic disease lie in their undergraduate and postgraduate education,while for patients with a psychotic disorder lie in their basic education**. **Lack of individuality, agency, and emotional response [1] leads patients to feel that their individual identities are degraded by a depersonalized medical practice [5] and respond with mechanistic dehumanization when physicians see them as mechanical systems with interacting parts [29]; according to this view, the disease can be explained as machine dysfunction [30].

Also, patients with the organic disease who have university education have more animalistic dehumanized than patients with the organic disease who are primary school graduates, while patients with the psychotic disorder who have a degree and elementary school graduates have more animalistic dehumanized than patients with the psychotic disorder who are high school graduates. It seems that both the examined groups, who have higher educatiοn, feel more like animals during their treatment and are dehumanized animalistically, probably due to their reduced autonomy and non-participation in decision-making, thus reacting in this way to the faceless hospital environment.

The educational level also affected the self-dehumanization of patients with the organic disease and patients with psychotic disorder in the present study. Specifically, patients with the organic disease who were elementary school graduates self-dehumanized more than patients with the organic disease who were graduates of the university, and patients with the psychotic disorder who were high school graduates self-dehumanized more than patients with the psychotic disorder who were university graduates. There were also differences in both the ratings of the human nature feature and the human uniqueness feature in patients with the organic disease and in patients with a psychotic disorder. Patients with the organic disease, who are graduates of the university, attribute more of the characteristic of human nature to themselves than patients with the organic disease who are graduates of primary school. Patients with a psychotic disorder who hold a master's/doctoral degree, university graduates, and elementary school graduates attribute the characteristic of human nature to themselves more than patients with the psychotic disorder who are high school graduates. In other words, high school graduates realize that they do not have a sense of common humanness (human nature) [4]. Regarding the attribution of the human uniqueness characteristics to themselves, the patients with the organic disease who are graduates of university attribute more the characteristics of human uniqueness to themselves than the patients with the organic disease who are primary school graduates. Patients with a psychotic disorder who are graduates of university attribute the characteristics of human uniqueness to themselves more than patients with the psychotic disorder who are graduates of high school. It seems that university and postgraduate education have a protective effect on self-dehumanization and the attribution of humanness to oneself, possibly due to greater self-esteem, greater flexibility, and the use of a more complex problem-solving process. It may also be related to the age of onset of the disease, which may later affect its severity, as it is probable that people with early onset of the disease have not been able to progress through the educational levels. In conclusion, the educational level of both patients with the organic disease and patients with psychotic disorder seems to affect both mechanistic and animalistic dehumanization and self-dehumanization.

Naturally, our research was subject to several limitations. First, patients with organic disease had many different diagnoses, while those with mental illness only had psychosis. Second, the sample consisted of 200 people, and it would be good to use a larger sample in the future. Third, the sample comes from a single hospital. For this reason, we believe that even better results could be obtained with a sample from different hospitals in different parts of the country. Finally, we must also keep in mind that samples consisting of patients with the organic disease and patients with a psychotic disorder are populations vulnerable to the given condition. More specifically, the majority of patients with a psychotic disorder are involuntarily hospitalized following a prosecutor's order and the majority of patients with the organic disease have serious health problems, so questions arise regarding the understanding of the questions that are posed in the measuring instruments.

## Conclusions

From the research findings, it is clear that dehumanization and self-dehumanization are phenomena that exist in the Greek hospital. Thus it is necessary to take measures to address them, in order to improve the quality of care provided and contribute to the well-being of employees, who are using the dehumanization of patients as a defense against occupational fatigue and health system deficiencies. It is important to take measures to train specialists in the hospital so that they can establish a safe therapeutic relationship with the patient. In this way, patients will not resort to dehumanization and self-dehumanization as a defense against the stress of hospitalization. Finally, the research was conducted before the appearance of COVID-19 and perhaps if the research was done today, the findings would be different as the patients are alone in the wards, without any supportive framework.
